# Moderate aortic stenosis identified during intensive care admission for a non-cardiac primary diagnosis and 90-day mortality: a retrospective cohort study using the MIMIC-IV echocardiography database

**DOI:** 10.1186/s12872-026-06468-3

**Published:** 2026-08-13

**Authors:** Lujun Shao, Cheng Xu, Huiyu Tai, Yue Li

**Affiliations:** 1 Department of Intensive Care Unit, The Affiliated Taizhou People’s Hospital of Nanjing Medical University, Taizhou, 225300 China; 2 Graduate School of Medicine, Hirosaki University, Hirosaki, 036-8562 Japan

**Keywords:** Aortic stenosis, Critical illness, Intensive care, Echocardiography, Mortality, MIMIC-IV

## Abstract

**Background:**

Moderate aortic stenosis is associated with reduced survival in ambulatory cardiology populations, but its prognostic significance when identified during an intensive care admission for a non-cardiac primary diagnosis is unknown. We examined its association with 90-day mortality in this setting.

**Methods:**

Retrospective cohort using the Medical Information Mart for Intensive Care IV (MIMIC-IV) version 3.1 and its echocardiogram-matched subset (MIMIC-IV-ECHO). We included each adult’s first intensive care admission for a non-cardiac primary diagnosis with an interpretable baseline transthoracic echocardiogram recording peak aortic jet velocity. Stenosis was graded from the peak velocity (no stenosis < 2.0, sclerosis or mild 2.0–2.9, moderate 3.0–3.9, severe ≥ 4.0 m/s). The echocardiogram defined time zero; the primary outcome was all-cause mortality within a fixed 90-day window. Associations were estimated with multivariable Cox models adjusted for acute-illness severity and comorbidity, with propensity-score matching, velocity–gradient concordance grading, pre-specified ejection-fraction subgroups, and an E-value.

**Results:**

Of 12,878 patients (10,511 no stenosis, 1,797 sclerosis, 411 moderate, 159 severe), 3,605 died within 90 days. Crude 90-day mortality rose across grades (26.1%, 34.4%, 40.4%, 46.5%). After adjustment, the hazard increased monotonically: sclerosis 1.17 (95% CI 1.07–1.28), moderate 1.33 (1.13–1.56), severe 1.61 (1.27–2.03). The moderate-stenosis association was modest but consistent, attenuating slightly in the most conservative specifications (propensity-score matched 1.20 [1.02–1.40]; parsimonious 1.22 [1.05–1.43]); the E-value was 1.73 (lower limit 1.41). It was numerically concentrated in the first two weeks (1.58 [1.30–1.93], days 0–14) and numerically larger with reduced (1.47 [1.10–1.97]) than preserved ejection fraction (1.10 [0.87–1.39]); neither the proportional-hazards nor the grade-by-ejection-fraction interaction test was significant (interaction *P* = 0.21), and both are exploratory numerical patterns, not demonstrated effect modification.

**Conclusions:**

In non-cardiac critical illness, velocity-defined moderate aortic stenosis identified during the intensive care admission was independently associated with higher 90-day mortality, with exploratory numerical patterns of greater early risk and of greater risk with reduced ejection fraction. These data identify a higher-risk phenotype; they do not establish a causal adverse effect of moderate valvular obstruction itself. It is best regarded as a prognostic marker warranting closer surveillance rather than, on current evidence, a target for valve intervention.

**Supplementary Information:**

The online version contains supplementary material available at https://doi.org/10.1186/s12872-026-06468-3.

## Background

Calcific aortic stenosis is among the most common forms of valvular heart disease in ageing populations, and its prevalence rises steeply with age [1, 2]. Echocardiography is the reference method for grading severity, integrating the peak aortic jet velocity, the mean transvalvular gradient, and the calculated aortic valve area [3]. Severe aortic stenosis, once it becomes symptomatic, carries a poor prognosis unless the valve is replaced, and current guidelines reserve valve intervention largely for severe disease [4, 5]. 

Clinical attention has recently extended to less-than-severe disease. Large observational studies have shown that moderate aortic stenosis is associated with substantially reduced survival [6, 7], and the excess risk appears particularly marked in patients with left-ventricular systolic dysfunction [8]. These observations have prompted interest in whether earlier valve intervention might benefit selected patients. Almost all of this evidence, however, comes from ambulatory cardiology populations in whom aortic stenosis was the focus of evaluation and severity was graded by comprehensive, flow-corrected echocardiography.

Critically ill patients differ in important ways. Echocardiography is performed frequently in the intensive care unit (ICU), usually to investigate haemodynamic instability rather than the aortic valve, so moderate stenosis is commonly documented during an admission undertaken for another reason. Two features make its prognostic interpretation uncertain in this setting. First, the peak aortic jet velocity—often the only grading parameter available in routine records—is flow-dependent and may be lowered by a reduced stroke volume or raised by the hyperdynamic states common in critical illness, so velocity-based grading can misclassify severity [3]. Second, whether a haemodynamically modest valve lesion documented in these circumstances carries prognostic weight beyond the acute illness and its comorbidities is unknown. To our knowledge, the prognostic significance of moderate aortic stenosis identified during an ICU admission for a non-cardiac primary diagnosis has not been quantified.

The recent release of structured echocardiographic measurements in the echocardiogram-matched subset of the Medical Information Mart for Intensive Care IV (MIMIC-IV) makes it feasible to address this question at scale. We therefore examined whether moderate aortic stenosis identified during an ICU admission for a non-cardiac primary diagnosis is associated with 90-day mortality, and whether any such association is graded across the spectrum of velocity-defined severity.

## Methods

### Data sources and linkage

We performed a retrospective cohort study using MIMIC-IV version 3.1 [9] and its echocardiographic companion module, the MIMIC-IV echocardiogram-matched subset (MIMIC-IV-ECHO) version 1.0 [10]; both are distributed through PhysioNet [11]. MIMIC-IV is a single-centre database of patients admitted to the intensive care units (ICUs) of the Beth Israel Deaconess Medical Center, Boston, Massachusetts. MIMIC-IV-ECHO provides structured transthoracic echocardiographic measurements—including the peak aortic jet velocity, the mean transvalvular gradient, and the left-ventricular ejection fraction—derived from studies recorded between 2008 and 2022. Because de-identified patient identifiers and the per-patient date-shift offsets are preserved across MIMIC-IV releases, echocardiographic studies were linked to hospitalizations and ICU stays at the patient and admission level. The study followed the Strengthening the Reporting of Observational Studies in Epidemiology (STROBE) reporting guideline [12]. 

### Study population

We identified the first ICU admission of each adult (≥ 18 years) with an interpretable baseline transthoracic echocardiogram that recorded a peak aortic jet velocity obtained between 24 h before and 48 h after ICU admission; the echocardiogram defined the index time (time zero) for follow-up. Patients with a previous surgical or transcatheter aortic valve replacement were excluded, because transprosthetic velocities do not reflect native disease. The primary cohort was restricted to patients whose primary reason for admission was a non-cardiac condition, so that aortic stenosis was identified on an echocardiogram obtained during the evaluation of the acute illness rather than being the reason for admission. Because pre-admission ambulatory records and prior outpatient echocardiograms are not captured in the database, we could not determine whether the valve lesion had already been diagnosed before the index admission. The exposure is therefore described throughout as aortic stenosis identified during an ICU admission for a non-cardiac primary diagnosis — a statement about the circumstances of detection, not a claim that the lesion was previously unknown or unrelated to the clinical presentation. A non-cardiac admission was defined as a primary hospital diagnosis outside the heart-specific categories (ischaemic heart disease, heart failure, cardiac arrhythmia, valvular and rheumatic heart disease, pericardial and myocardial disease, and cardiac arrest); cerebrovascular and other vascular diagnoses were classified as non-cardiac. Patients who died or were censored on or before the index time and those with incomplete covariate data were excluded from the complete-case analysis. The broader cohort of all critically ill adults, including those admitted for cardiac conditions, was retained for a sensitivity analysis.

### Exposure: grading of aortic stenosis

Aortic stenosis severity was graded from the peak aortic jet velocity according to the American Society of Echocardiography and the European Association of Cardiovascular Imaging [3]: no stenosis (< 2.0 m/s), aortic sclerosis or mild stenosis (2.0–2.9 m/s; hereafter the aortic-sclerosis reference group), moderate stenosis (3.0–3.9 m/s), and severe stenosis (≥ 4.0 m/s). Velocities outside 0.5–8.0 m/s were treated as implausible and set to missing. Because the aortic valve area, the left-ventricular outflow-tract velocity–time integral, and the dimensionless index were not available in the database, flow-independent grading could not be performed; the peak velocity is flow-dependent and may be raised by high-output states or lowered by a reduced stroke volume. To mitigate this, the mean transvalvular gradient—recorded in a subset of studies—was used to grade severity concurrently in a concordance analysis, and variables reflecting the systemic circulatory state (heart rate, temperature, and haemoglobin concentration) were included as covariates. These variables are indirect surrogates for the circulatory conditions that raise or lower transvalvular velocity; they are not measurements of transvalvular flow itself, which would require the stroke volume or the left-ventricular outflow-tract velocity–time integral, and they therefore mitigate but cannot eliminate flow-dependent misclassification.

### Covariates

Covariates were specified a priori as determinants of mortality in critical illness and potential confounders of the association between aortic stenosis and death. They comprised age, sex, the first-day Sequential Organ Failure Assessment (SOFA) score, the Charlson comorbidity index, individual comorbidities (hypertension, atrial fibrillation, diabetes mellitus, and chronic kidney disease), receipt of vasopressors and invasive mechanical ventilation on the first ICU day, and three indirect markers of the systemic circulatory state intended to mitigate flow-dependent misclassification of stenosis severity—the first-day minimum haemoglobin concentration, mean heart rate, and maximum temperature; these are surrogates for conditions that alter transvalvular flow rather than direct measures of flow. Full definitions of all variables, data sources, and analytic details are provided in Additional file 1: Supplementary Methods and Table S5.

### Outcomes

Follow-up was anchored at the baseline echocardiogram, which defined time zero. The primary outcome was all-cause mortality within 90 days of that echocardiogram, analysed over a fixed follow-up window that did not require a minimum length of stay, thereby avoiding the immortal-time and survivor biases that arise when follow-up is conditioned on survival to a landmark [13]. Secondary outcomes were in-hospital mortality, with follow-up censored at hospital discharge, and 28-day mortality.

### Statistical analysis

The analytic strategy comprised multivariable adjustment as the primary approach, complemented by propensity-score matching, a velocity–gradient concordance analysis to address flow-dependent misclassification of severity, and a quantitative sensitivity analysis for unmeasured confounding. Baseline characteristics are summarized as medians with interquartile ranges for continuous variables and counts with percentages for categorical variables, compared across stenosis grades with the Kruskal–Wallis and chi-square tests. The primary analysis was a multivariable Cox proportional-hazards model relating stenosis grade (no stenosis as the reference) to 90-day mortality, adjusted for all covariates; the proportional-hazards assumption was assessed with scaled Schoenfeld residuals and discrimination with Harrell’s concordance statistic. The dose–response relationship between peak velocity and mortality was modelled with restricted cubic splines (knots at the 5th, 35th, 65th, and 95th percentiles) adjusted for the same covariates. Secondary outcomes were analysed with a Cox model (in-hospital mortality, censored at discharge) and logistic regression (28-day and 90-day mortality).

To complement covariate adjustment, moderate stenosis was compared with no stenosis after 1:4 nearest-neighbour propensity-score matching without replacement on the logit of the propensity score, using a calliper of 0.2 standard deviations; the matched hazard ratio was estimated with a Cox model using cluster-robust standard errors, and covariate balance was assessed with standardized mean differences [14]. To probe the validity of velocity-based grading and the robustness of the findings, we performed several pre-specified analyses: re-grading moderate and severe stenosis by concurrent peak-velocity and mean-gradient criteria; restriction to a non-hyperdynamic subset (heart rate ≤ 100 beats/min, temperature ≤ 38 °C, and haemoglobin ≥ 9 g/dL), that is, to patients free of the tachycardic, febrile, or anaemic states that raise transvalvular velocity; restriction to echocardiograms performed within 24 h of admission; refitting of the primary model without the vasopressor and mechanical-ventilation terms; and extension of the analysis to the full cohort including cardiac admissions. Because heart failure and a reduced ejection fraction may confound the association but may also lie on the causal pathway from aortic stenosis to death, they were excluded from the primary model and examined only in sensitivity analyses. To address potential over-adjustment and residual confounding, we additionally fitted a parsimonious model adjusted only for age, sex, the SOFA score, and the Charlson index, a model adding a heart-failure term, and a model further adding the left-ventricular ejection fraction. Pre-specified subgroup analyses were conducted in patients with preserved (≥ 50%) and reduced (< 50%) left-ventricular ejection fraction, restricted to those in whom ejection fraction was recorded, and the difference between strata was assessed with a multiplicative stenosis-grade-by-ejection-fraction interaction term tested by likelihood ratio. This test was interpreted as the formal assessment of effect modification, with the stratum-specific estimates regarded as descriptive. The robustness of the moderate-stenosis association to unmeasured confounding was quantified with the E-value, computed for a common outcome [15]. Subgroup and sensitivity analyses were exploratory and were not adjusted for multiple comparisons. Missing covariate data affected fewer than 3% of eligible patients and were handled by complete-case analysis. Analyses were performed in R version 4.5.2, using the packages listed in Additional file 1: Supplementary Methods. Two-sided P values below 0.05 were considered statistically significant.

## Results

### Cohort and baseline characteristics

**Fig. 1:**
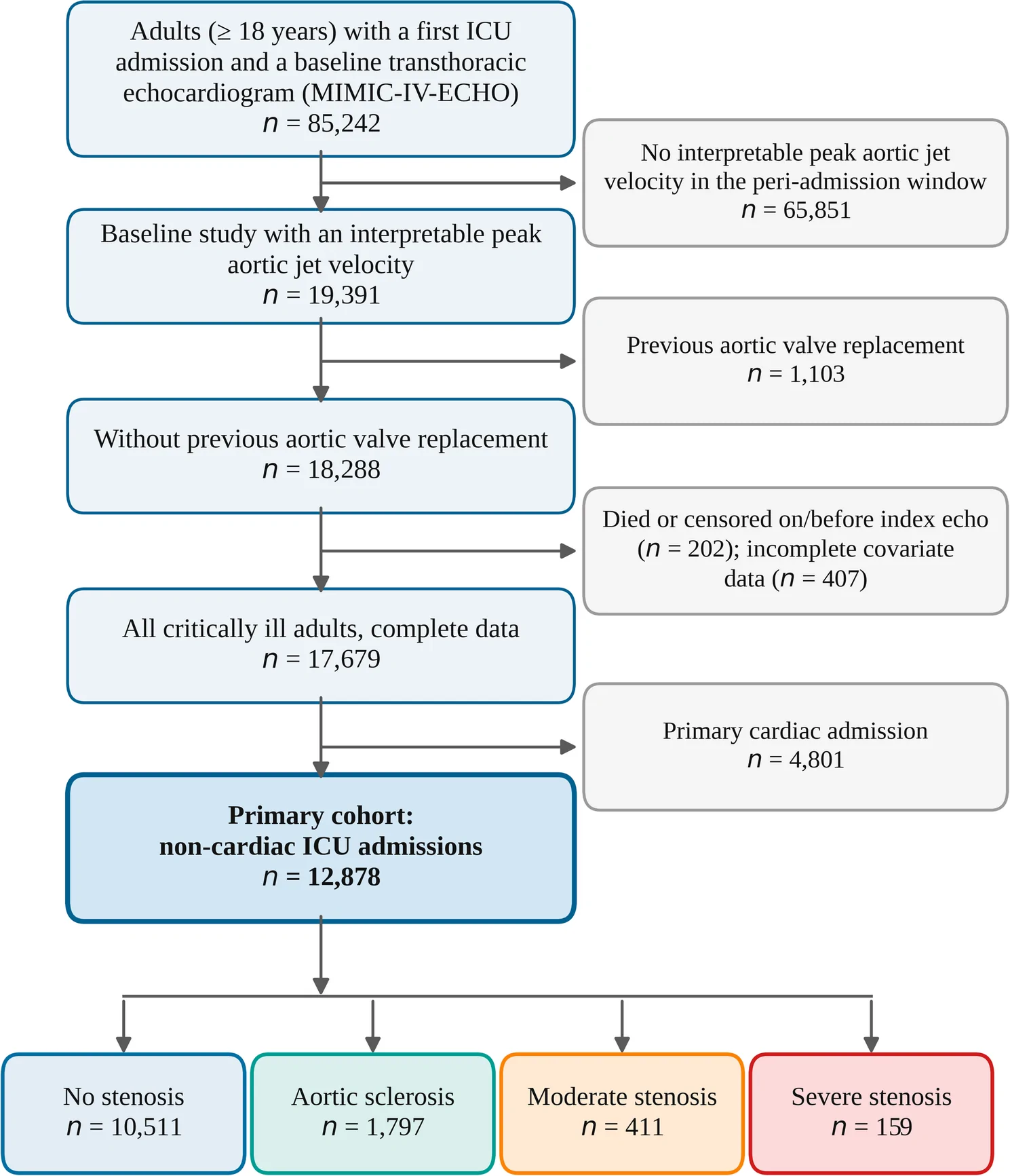
Selection of the primary cohort of adults admitted to intensive care for a non-cardiac condition. From 85,242 first ICU admissions of adults, 65,851 without an interpretable baseline aortic peak velocity and 1,103 with a previous aortic valve replacement were excluded, leaving 18,288; a further 202 without follow-up beyond the index echocardiogram and 407 with incomplete covariate data were excluded (17,679), and 4,801 admitted for a cardiac primary diagnosis were excluded, yielding 12,878 patients graded as no stenosis (*n* = 10,511), aortic sclerosis (*n* = 1,797), moderate stenosis (*n* = 411), or severe stenosis (*n* = 159)

Among 85,242 adults in their first ICU admission, 65,851 had no interpretable baseline aortic peak velocity and 1,103 had a previous aortic valve replacement, leaving 18,288 patients; a further 202 with no follow-up beyond the index echocardiogram and 407 with incomplete covariate data were excluded (17,679 patients), and 4,801 admitted for a cardiac primary diagnosis were excluded, yielding 12,878 patients in the primary cohort (Fig. 1). Of these, 10,511 had no stenosis, 1,797 aortic sclerosis, 411 moderate stenosis, and 159 severe stenosis. Increasing stenosis grade was accompanied by older age (median 67 to 82 years) and a higher burden of chronic disease—atrial fibrillation (33% to 55%), heart failure (37% to 62%), chronic kidney disease (26% to 43%, peaking in the moderate-stenosis group), and the Charlson index (median 5 to 7)—whereas differences in acute-illness severity across grades were modest in absolute terms, though several were statistically significant in this large sample (SOFA score median 4 to 5 and vasopressor use 28% to 35%, both *P* < 0.001; mechanical ventilation 28% to 35% without a consistent gradient, being lowest in the severe-stenosis group, *P* = 0.12) (Table 1). A mean transvalvular gradient, available in 2,003 patients (15.6%) and recorded more often at higher stenosis grades, rose monotonically across grades (median 5, 12, 25, and 47 mmHg), corroborating the velocity-based classification. Over 90 days there were 3,605 deaths, of which 2,099 (58.2%) occurred in hospital and 1,506 (41.8%) after discharge (the 2,116 in-hospital deaths shown in Table 1 include 17 occurring more than 90 days after the echocardiogram); the fixed follow-up window therefore captured deaths beyond the index hospitalization.

**Table 1:** Baseline characteristics by aortic stenosis grade in the primary non-cardiac cohort (*n* = 12,878)

Characteristic	No stenosis (*n* = 10,511)	Aortic sclerosis (*n* = 1,797)	Moderate stenosis (*n* = 411)	Severe stenosis (*n* = 159)	*P* value
*Demographics*					
Age, years	67.0 [56.0–78.0]	73.0 [62.0–83.0]	80.0 [71.0–87.0]	82.0 [75.0–89.0]	< 0.001
Female sex	4909 (46.7)	946 (52.6)	193 (47.0)	78 (49.1)	< 0.001
*Acute illness severity and organ support*					
SOFA score	4.0 [2.0–7.0]	5.0 [3.0–8.0]	5.0 [3.0–8.0]	5.0 [3.0–7.5]	< 0.001
Vasopressor, day 1	2898 (27.6)	546 (30.4)	145 (35.3)	55 (34.6)	< 0.001
Mechanical ventilation, day 1	3494 (33.2)	634 (35.3)	130 (31.6)	44 (27.7)	0.12
*Comorbidity burden*					
Charlson comorbidity index	5.0 [3.0–8.0]	6.0 [5.0–8.0]	7.0 [6.0–9.0]	7.0 [5.0–8.0]	< 0.001
Hypertension	7001 (66.6)	1377 (76.6)	331 (80.5)	122 (76.7)	< 0.001
Atrial fibrillation	3440 (32.7)	704 (39.2)	219 (53.3)	88 (55.3)	< 0.001
Diabetes mellitus	3382 (32.2)	699 (38.9)	168 (40.9)	49 (30.8)	< 0.001
Chronic kidney disease	2724 (25.9)	652 (36.3)	175 (42.6)	66 (41.5)	< 0.001
Heart failure	3847 (36.6)	815 (45.4)	254 (61.8)	99 (62.3)	< 0.001
Chronic pulmonary disease	2945 (28.0)	586 (32.6)	134 (32.6)	56 (35.2)	< 0.001
*Indirect markers of circulatory state (first ICU day)*					
Haemoglobin, minimum, g/dL	10.3 [8.5–12.0]	9.4 [8.0–11.0]	9.4 [8.1–10.8]	9.6 [8.1–11.3]	< 0.001
Heart rate, mean, /min	85.8 [74.1–98.5]	83.6 [72.1–95.5]	81.6 [71.0–94.5]	80.3 [71.6–91.8]	< 0.001
Temperature, maximum, °C	37.2 [36.9–37.7]	37.2 [36.9–37.7]	37.2 [36.9–37.7]	37.1 [36.9–37.4]	0.011
*Echocardiographic findings*					
Peak aortic velocity, m/s	1.4 [1.2–1.6]	2.2 [2.1–2.5]	3.3 [3.1–3.6]	4.4 [4.2–4.8]	< 0.001
Mean transvalvular gradient, mmHg	5.0 [4.0–7.0]	12.0 [10.0–15.0]	25.0 [22.0–31.0]	47.0 [43.0–56.0]	< 0.001
Left-ventricular ejection fraction, %	55.0 [50.0–63.0]	60.0 [55.0–70.0]	55.0 [40.0–65.0]	59.0 [45.0–65.0]	< 0.001
Preserved ejection fraction (≥ 50%)	6282 (59.8)	1218 (67.8)	243 (59.1)	98 (61.6)	< 0.001
*Length of stay and mortality*					
ICU stay, days	2.7 [1.5–5.5]	3.0 [1.7–5.9]	2.7 [1.5–5.1]	2.2 [1.5–3.9]	< 0.001
Hospital stay, days	8.0 [5.0–15.0]	9.0 [5.0–16.0]	8.0 [5.0–13.0]	7.0 [4.0–12.0]	< 0.001
In-hospital death	1601 (15.2)	378 (21.0)	100 (24.3)	37 (23.3)	< 0.001
28-day death	2001 (19.0)	477 (26.5)	128 (31.1)	56 (35.2)	< 0.001
90-day death	2747 (26.1)	618 (34.4)	166 (40.4)	74 (46.5)	< 0.001

Values are median [interquartile range] for continuous variables and n (%) for categorical variables. *P* values are from the Kruskal–Wallis test (continuous) or chi-square test (categorical) across the four stenosis grades. Aortic stenosis grade was assigned from the peak aortic jet velocity (no stenosis < 2.0, aortic sclerosis 2.0–2.9, moderate 3.0–3.9, severe ≥ 4.0 m/s). A mean transvalvular gradient was recorded in 2,003 of the 12,878 patients (no stenosis 380, aortic sclerosis 1,076, moderate 389, severe 158). Left-ventricular ejection fraction was recorded in 10,324 patients (80.2%); preserved-ejection-fraction percentages are expressed relative to the full group in each column. ICU, intensive care unit; SOFA, Sequential Organ Failure Assessment

### Primary analysis: 90-day mortality

**Fig. 2:**
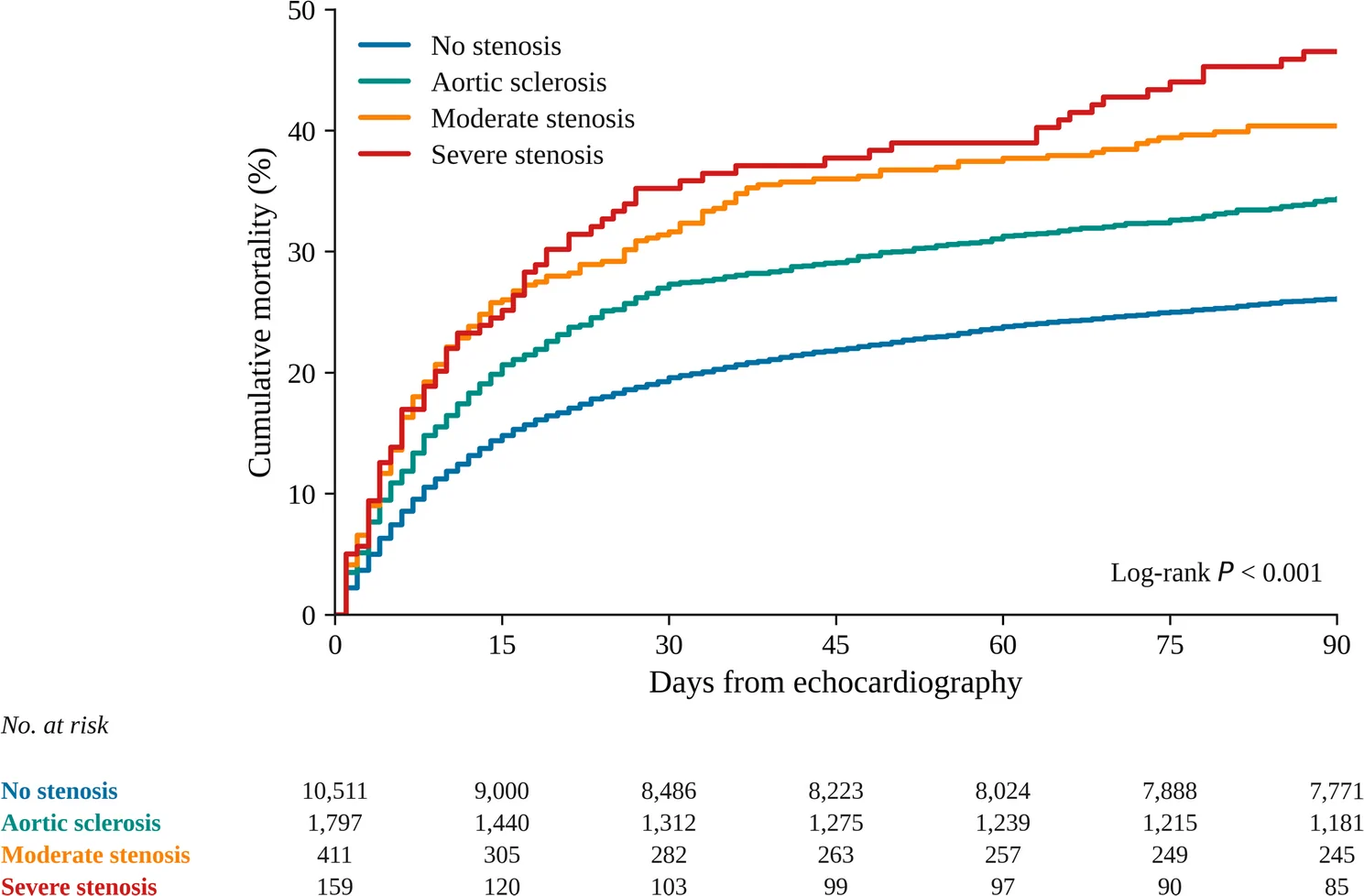
Cumulative 90-day mortality (1 − Kaplan–Meier survival) by aortic-stenosis grade in the primary (non-cardiac) cohort. Mortality rose progressively with increasing velocity-based grade; the difference across grades was significant (log-rank *P* < 0.001). Numbers of patients at risk are shown below the time axis

Crude 90-day mortality increased across stenosis grades (26.1%, 34.4%, 40.4%, and 46.5%). In the multivariable Cox model, the adjusted hazard of death within 90 days rose with stenosis severity relative to no stenosis: aortic sclerosis 1.17 (95% confidence interval [CI] 1.07–1.28), moderate stenosis 1.33 (1.13–1.56), and severe stenosis 1.61 (1.27–2.03) (Table 2; Additional file 1: Table S1). Discrimination was acceptable (Harrell’s concordance statistic 0.76, 95% CI 0.75–0.77). The proportional-hazards assumption was not rejected for aortic stenosis grade (Schoenfeld test, *P* = 0.32), but the time-course of risk differed by grade: in a post-hoc time-stratified analysis the moderate-stenosis hazard was numerically concentrated early (1.58 [1.30–1.93] in days 0–14 versus 1.04 [0.80–1.36] in days 15–90), whereas severe stenosis carried a sustained, later hazard (1.51 versus 1.69); these opposing trends across grades offset one another, so the overall test was not significant (Additional file 1: Table S4). The 90-day moderate-stenosis hazard ratio is therefore best interpreted as a period-averaged estimate over an interval in which the hazard was numerically front-loaded, consistent with the larger in-hospital estimate below. Kaplan–Meier curves separated progressively by grade (Fig. 2). The E-value for the moderate-stenosis hazard ratio, computed for a common outcome, was 1.73 (lower confidence limit 1.41), meaning that an unmeasured confounder would need to be associated with both moderate stenosis and mortality by a risk ratio of about 1.7, beyond the measured covariates, to explain away the association.

**Table 2:** Adjusted associations of aortic stenosis grade with mortality in the primary (non-cardiac) cohort

Comparison (vs. no stenosis)	Measure	Aortic sclerosis (95% CI)	Moderate stenosis (95% CI)	Severe stenosis (95% CI)
*Primary outcome*				
90-day mortality	HR	1.17 (1.07–1.28)	1.33 (1.13–1.56)	1.61 (1.27–2.03)
*Alternative outcomes and estimators*				
In-hospital mortality	HR	1.21 (1.08–1.35)	1.54 (1.25–1.89)	1.60 (1.15–2.22)
28-day mortality	OR	1.26 (1.11–1.44)	1.46 (1.15–1.85)	1.90 (1.33–2.73)
90-day mortality	OR	1.17 (1.04–1.32)	1.35 (1.08–1.69)	1.93 (1.37–2.73)
90-day mortality, propensity-score matched	HR	—	1.20 (1.02–1.40)	—
*Concordant velocity–gradient grading*				
90-day mortality	HR	—	1.36 (1.15–1.61)	1.63 (1.27–2.09)

Estimates are adjusted for all covariates (see Methods), with no stenosis as the reference; em dashes denote categories not estimated for a given analysis. Hazard ratios (HR) are from Cox proportional-hazards models and odds ratios (OR) from logistic regression. The proportional-hazards assumption was not rejected for aortic stenosis grade (Schoenfeld test, *P* = 0.32); on post-hoc time-stratified analysis the moderate-stenosis hazard was numerically concentrated in the first 14 days (Additional file 1: Table S4), and the in-hospital and 90-day odds-ratio estimates corroborate the association. Propensity-score matching paired 411 patients with moderate stenosis to 1,644 with no stenosis (1:4 nearest-neighbour without replacement, calliper 0.2 standard deviations of the logit; 2,055 patients; all 411 moderate-stenosis patients were matched, and all standardized mean differences were < 0.05). Concordant grading re-classified moderate and severe stenosis by concurrent peak-velocity and mean-gradient criteria against the full no-stenosis reference. The full multivariable model, matching balance, and concordance details are provided in Additional file 1: Tables S1–S4. CI, confidence interval

### Dose–response relationship

**Fig. 3:**
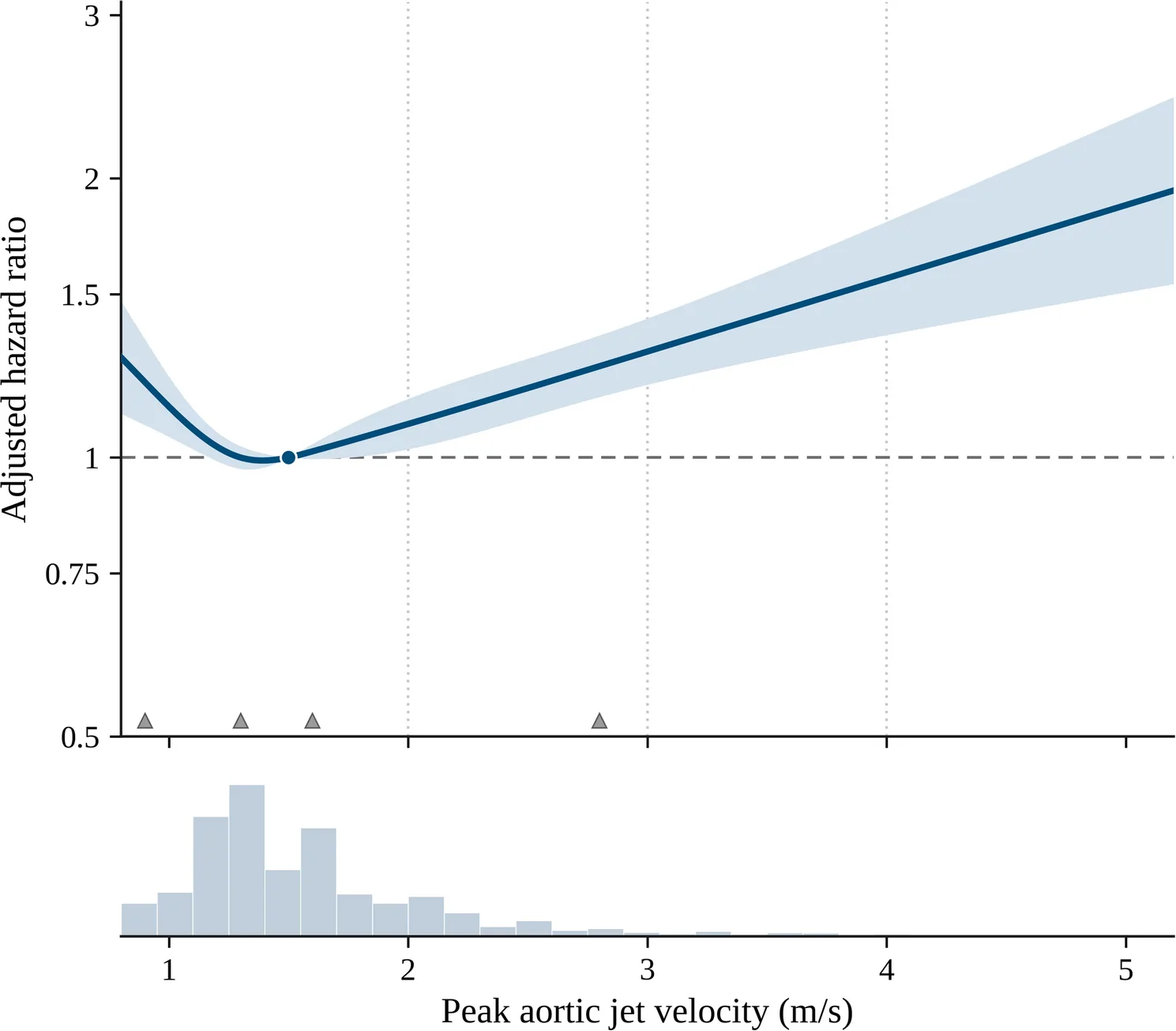
Adjusted dose–response between peak aortic jet velocity and 90-day mortality. Restricted cubic spline (knots at the 5th, 35th, 65th, and 95th percentiles of peak velocity) from a Cox model with full covariate adjustment; the solid line is the adjusted hazard ratio and the shaded band its 95% confidence interval, plotted relative to the cohort median peak velocity (1.5 m/s, filled circle). Knot positions are marked by triangles, grade thresholds (2, 3 and 4 m/s) by dotted lines, and the distribution of peak velocity is shown below. The association was significantly nonlinear (*P* < 0.001): the adjusted hazard was lowest near the median velocity and rose monotonically at higher velocities, with wide confidence intervals above the highest knot (≈ 2.8 m/s) where data were sparse; a modest upturn was also present at the lowest velocities

Modelled continuously with restricted cubic splines and full covariate adjustment, peak aortic velocity showed a significant overall (χ² = 45.8, 3 degrees of freedom, *P* < 0.001) and nonlinear (χ² = 24.5, 2 degrees of freedom, *P* < 0.001) association with 90-day mortality. The adjusted hazard reached its nadir near the cohort median velocity (approximately 1.5 m/s) and rose monotonically thereafter; data were sparse and confidence intervals correspondingly wide above the highest knot (approximately 2.8 m/s, the 95th percentile), so the high-velocity portion of the curve reflects extrapolation. A modest upturn in the adjusted hazard was also apparent at the lowest velocities (below approximately 1.3 m/s), consistent with the elevated risk of low-output, low-flow states (Fig. 3).

### Alternative outcomes and estimators

The association was consistent across outcomes and estimators (Table 2). For in-hospital mortality the adjusted hazard ratio for moderate stenosis was 1.54 (1.25–1.89); in a Fine-Gray competing-risks analysis treating live discharge as a competing event, the subdistribution hazard ratio for moderate stenosis was 1.47 (1.19–1.81), similar to this cause-specific estimate and indicating that the in-hospital association was not an artefact of informative censoring at discharge (Additional file 1: Table S4). The adjusted odds ratio was 1.46 (1.15–1.85) at 28 days and 1.35 (1.08–1.69) at 90 days. The larger in-hospital estimate is consistent with the front-loaded hazard noted above. Severe stenosis showed correspondingly larger estimates (28-day odds ratio 1.90, 1.33–2.73; 90-day odds ratio 1.93, 1.37–2.73). Because death was common in this cohort, these odds ratios overestimate the corresponding risk ratios and are reported only in support of the hazard-ratio analyses.

### Propensity-score matching

After 1:4 propensity-score matching of moderate stenosis to no stenosis (2,055 patients; all standardized mean differences < 0.05; Additional file 1: Table S2), moderate stenosis remained associated with 90-day mortality (hazard ratio 1.20, 1.02–1.40), consistent with the covariate-adjusted estimate.

### Velocity–gradient concordance

Among patients with velocity-defined moderate stenosis and a recorded mean gradient, 88% (341 of 389) were concordant by gradient (Additional file 1: Table S3). When moderate and severe stenosis were re-graded by concurrent velocity and gradient criteria, the moderate-stenosis hazard ratio was 1.36 (1.15–1.61) against the full no-stenosis reference and 1.29 (1.00–1.67) within the concordantly graded subset that had a recorded gradient, and the severe-stenosis hazard ratio was 1.63 (1.27–2.09) (Table 2; Additional file 1: Table S4).

### Sensitivity and subgroup analyses

**Fig. 4:**
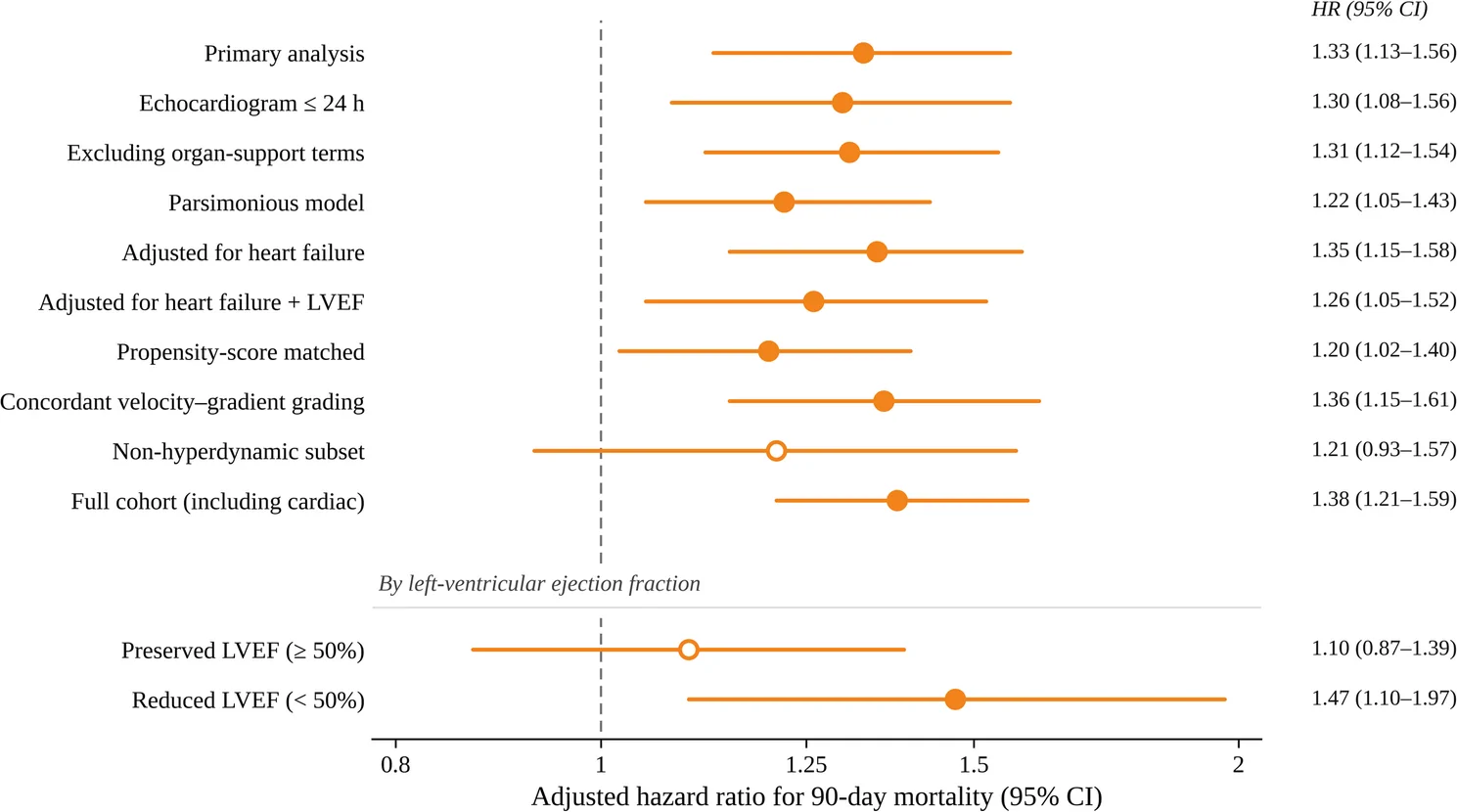
Adjusted hazard ratios for 90-day mortality with moderate aortic stenosis across sensitivity and subgroup analyses. Estimates are for moderate stenosis relative to no stenosis, in the primary analysis and in pre-specified sensitivity and subgroup analyses. Filled circles denote *P* < 0.05 and open circles a confidence interval that includes 1. Corresponding estimates for severe stenosis and full numerical results are reported in Table 2 and Additional file 1: Table S4

The moderate-stenosis association was broadly robust but heterogeneous (Fig. 4; Additional file 1: Table S4). It was similar when the analysis was restricted to echocardiograms obtained within 24 h of admission (1.30, 1.08–1.56), when the vasopressor and mechanical-ventilation terms were removed (1.31, 1.12–1.54), and when the cohort was extended to all critically ill adults including cardiac admissions (1.38, 1.21–1.59). In the non-hyperdynamic subset the moderate-stenosis estimate remained elevated but lost statistical significance (1.21, 0.93–1.57), most plausibly because this subset contained only 62 moderate-stenosis deaths; the same restriction applied to the full cohort, which retained more events, was significant (1.38, 1.12–1.71), and severe stenosis remained associated within the non-hyperdynamic subset (1.72, 1.20–2.49). The association was also unchanged in a parsimonious model adjusted only for age, sex, SOFA score, and Charlson index (1.22, 1.05–1.43), after adding a heart-failure term (1.35, 1.15–1.58), and after additionally adjusting for left-ventricular ejection fraction (1.26, 1.05–1.52), indicating that it was not an artefact of over-adjustment or of residual confounding by heart failure. Across ejection-fraction strata (the 10,324 patients with a recorded ejection fraction), the moderate-stenosis estimate was numerically larger in patients with reduced ejection fraction (1.47, 1.10–1.97) than in those with preserved ejection fraction (1.10, 0.87–1.39), whereas severe stenosis was associated in both; the same pattern was present in the full cohort (reduced ejection fraction 1.64, preserved 1.11). These subgroup estimates were compared with a formal multiplicative interaction test, which was not significant (*P* = 0.21 for the stenosis-grade-by-ejection-fraction interaction, 3 degrees of freedom), and their confidence intervals overlap substantially. The stratum-specific difference is therefore an exploratory numerical pattern: the data neither establish nor exclude effect modification by ejection fraction, and the pattern should not be read as demonstrating that moderate stenosis is harmful only when systolic function is impaired. It may nonetheless reflect that peak velocity underestimates severity under the low transvalvular flow accompanying a reduced ejection fraction—so that some truly severe stenosis is graded as moderate in this stratum—together with limited power in the preserved-ejection-fraction stratum.

## Discussion

In a large cohort of adults admitted to the intensive care unit for a non-cardiac primary diagnosis, velocity-defined aortic stenosis identified during that admission showed a graded association with 90-day mortality. After adjustment for the severity of the acute illness and for comorbidities, moderate stenosis was independently associated with death (hazard ratio 1.33), with a slightly smaller estimate in the more conservative propensity-matched and parsimonious specifications (1.20–1.22); the association is therefore best characterized as modest in magnitude. The gradient across aortic sclerosis, moderate, and severe disease was monotonic, and the association persisted after propensity-score matching and when severity was graded concordantly by the mean transvalvular gradient. Two exploratory features qualified this overall signal. The excess hazard associated with moderate stenosis was numerically concentrated in the first two weeks after the echocardiogram, and was numerically larger in patients with reduced ejection fraction, being small in those with preserved function. Neither pattern was supported by a significant formal test, and both are reported as exploratory rather than as established characteristics of the association.

These findings extend to the critically ill a body of evidence, derived almost entirely from ambulatory and referral populations, that links moderate aortic stenosis to reduced survival. Cohort studies and registries have repeatedly reported excess mortality in moderate disease and worse outcomes among patients followed in valve clinics [6, 7, 16], a meta-analysis of reconstructed time-to-event data from more than 400 000 patients estimated a large long-term survival deficit [17], and a very large echocardiographic cohort showed that the increased risk is largely independent of comorbidity and treatment [18]. A separate meta-analysis of interventional cohorts reached concordant conclusions [19]. Our adjusted hazard ratio of 1.33 is of comparable magnitude, indicating that the association is not abolished when stenosis is identified during an admission undertaken for a non-cardiac reason, although in our cohort the absolute risk is dominated by the acute illness itself. The more parsimonious and propensity-matched analyses yielded slightly lower estimates (hazard ratios 1.20–1.22), so the association is best summarized as modest but consistent across model specifications. Because a substantial proportion of moderate stenosis progresses to severe disease within a few years [20, 21], and asymptomatic severe disease itself carries a high event rate once it develops [22], part of the long-term excess reported elsewhere reflects progression to severe disease that our 90-day window could not capture.

The numerically larger estimate in patients with reduced ejection fraction is consistent with previous observations, but the between-stratum difference did not reach statistical significance on a formal interaction test (*P* = 0.21) and must be read as an exploratory numerical pattern rather than as confirmed effect modification. It nonetheless raises a clinically relevant possibility: that the risk observed here reflects the interaction between a moderate valvular obstruction and a vulnerable myocardium with limited functional reserve, rather than the effect of moderate stenosis alone. Moderate stenosis carries a particularly poor prognosis when left-ventricular systolic function is impaired [8], the importance of ejection fraction and symptoms in moderate disease has been emphasized [23], a reduced ejection fraction is itself a powerful and frequently under-recognized marker of risk both in aortic stenosis and in the wider population [24, 25], and relief of the valve obstruction has been associated with improved survival in patients who have stenosis together with ventricular dysfunction [26]. Two mechanisms are plausible. A poorly contracting ventricle tolerates the added afterload of even a moderate obstruction less well, so the haemodynamic cost of the lesion is greater. In addition, when transvalvular flow is low the peak velocity underestimates anatomical severity, so some patients graded as moderate by velocity in fact have more advanced, low-flow low-gradient disease.

This last point bears directly on a limitation intrinsic to our exposure. Guideline grading integrates velocity, gradient, and valve area [3], whereas we graded severity from the peak velocity alone in most patients. Discordance among velocity-, gradient-, and area-based grading is common in moderate aortic stenosis and marks subgroups with distinct prognoses [27], so velocity-only grading inevitably misclassifies a minority of patients. Reassuringly, the estimates were stable when severity was re-graded by the mean gradient in the subset with concordant data and when variables reflecting the systemic circulatory state were included as covariates; those variables (heart rate, temperature, and haemoglobin) are indirect surrogates rather than measures of transvalvular flow, so they mitigate rather than remove flow-dependent misclassification, and the corresponding restricted analysis is better described as a non-hyperdynamic than as a low-flow subset; because the mean gradient is itself derived from the transvalvular velocity, however, this concordance reflects internal consistency of the Doppler measurements rather than independent, flow-independent confirmation of severity, which would require the aortic valve area or the dimensionless index. Residual misclassification in low-flow states—most relevant when the ejection fraction is reduced, in whom some velocity-defined moderate stenosis may in truth be low-flow severe disease—could therefore contribute to the stronger estimate in that subgroup, alongside a genuine difference in ventricular tolerance.

The apparent early concentration of risk in moderate stenosis—derived from a post-hoc, time-stratified analysis and therefore hypothesis-generating—is nonetheless clinically coherent: in severe stenosis the excess hazard was sustained across the 90 days, whereas in moderate stenosis it was numerically concentrated in the first two weeks, when the limited cardiac reserve imposed by even a moderate obstruction is least able to meet the demands of acute illness. The prognosis of aortic stenosis is increasingly understood to depend not on valve severity alone but on the extent of associated cardiac damage [28–30], and the echocardiographic abnormalities that accompany moderate stenosis carry independent prognostic weight [31]. Consistent with this, the natural history of moderate stenosis differs markedly between preserved and reduced ejection fraction [32]. Our data are compatible with the hypothesis that, in the acute setting, it is the interaction between a modest fixed obstruction and a vulnerable or failing ventricle, rather than the valve lesion in isolation, that carries the short-term risk. This remains a hypothesis: the present design can identify the phenotype but cannot isolate the contribution of the valve lesion itself.

Clinically, these results identify velocity-defined moderate aortic stenosis found during a non-cardiac ICU admission as a marker of higher short-term risk rather than, on present evidence, a treatment target in the critically ill, in keeping with guidelines that reserve valve intervention for severe disease [5]. The finding could nonetheless prompt closer monitoring during the index illness and structured cardiology follow-up after recovery, particularly when the ejection fraction is reduced. Whether valve intervention alters outcome in moderate stenosis is being tested directly: a randomized trial of transcatheter replacement in heart failure with reduced ejection fraction and moderate stenosis did not meet its primary endpoint but improved quality of life [33], randomized trials of pre-emptive valve replacement in asymptomatic severe disease have reduced some adverse outcomes [34, 35], and the broader move toward earlier intervention in aortic stenosis continues to be evaluated [36]. Our observational data do not address treatment, cannot establish that moderate valvular obstruction itself causes the excess mortality, and should not be read as supporting valve intervention for moderate stenosis identified in this setting.

This study has several strengths. It is large and rests on quantitative echocardiographic measurements linked to granular intensive-care data [10], rather than on diagnostic codes, and it provides, to our knowledge, the first quantification of this association in adults admitted to intensive care for non-cardiac conditions. Anchoring follow-up at the echocardiogram and analysing a fixed 90-day window avoided the immortal-time and survivor biases that affect analyses conditioned on survival to a landmark [13], and the exposure–outcome association was probed with complementary approaches—propensity-score matching [14], concordant gradient-based grading, time-stratified models, and a quantitative sensitivity analysis for unmeasured confounding using the E-value [15]. 

Several limitations temper interpretation. First, the data derive from a single tertiary-care centre, and case mix, echocardiographic practice, and outcomes may differ elsewhere; we were unable to validate the findings externally, because in the available multicentre intensive-care database aortic stenosis is identifiable only from diagnostic codes, which cannot grade severity and which concentrate in cardiac rather than non-cardiac admissions, precluding a valid replication of a velocity-graded moderate-stenosis cohort identified during non-cardiac admissions. Second, as noted, severity was graded predominantly from the peak velocity [3], without the valve area, the dimensionless index, or any direct measure of transvalvular flow such as the stroke-volume index. The covariates used to mitigate this—heart rate, temperature, and haemoglobin—are indirect surrogates for the systemic circulatory state rather than measures of flow, so flow-dependent misclassification is reduced but not excluded, both in low-flow and in hyperdynamic conditions. Third, echocardiography was performed at the discretion of the treating team, usually to investigate haemodynamic instability [37], so the cohort comprises critically ill patients selected for having an early interpretable transthoracic study—roughly three-quarters of screened admissions lacked one—and the findings apply to this population rather than to all intensive-care patients. Here, non-cardiac denotes the primary reason for admission rather than the absence of cardiac disease: many patients had heart failure (62% of the moderate-stenosis group) or atrial fibrillation (53%). Moreover, because the database captures only care delivered at the study centre, we could not ascertain from prior diagnoses or prior echocardiograms whether aortic stenosis had been recognized before the index admission, nor whether it contributed to the presentation. We therefore describe the exposure only as stenosis identified during an ICU admission for a non-cardiac primary diagnosis, and make no claim that it was previously unknown or clinically unrelated; underlying cardiac disease may contribute to the observed association as residual confounding despite adjustment for heart failure in sensitivity analyses. Fourth, the study is observational; despite extensive adjustment, residual and unmeasured confounding cannot be excluded, and the modest E-value indicates that the moderate-stenosis association could be explained by an unmeasured confounder of moderate strength, so the relationship should be regarded as an association rather than a causal effect. Fifth, vital status after discharge relied on linked death records, and although the 90-day window falls within the period of reliable ascertainment, incomplete capture of out-of-hospital deaths cannot be excluded. Sixth, the in-hospital secondary outcome treats discharge as a censoring event; because discharge is informative, we also fitted a Fine-Gray subdistribution-hazard model with live discharge as a competing event [38], which gave a similar estimate for moderate stenosis (Additional file 1: Table S4), but this secondary outcome should nonetheless be interpreted more cautiously than the primary 90-day analysis. Finally, the moderate-stenosis association, although consistent across multiple specifications, was modest in magnitude and clinically heterogeneous—numerically front-loaded in time and numerically larger in patients with reduced ejection fraction, although neither pattern reached statistical significance on formal testing—and is therefore most useful for identifying a higher-risk phenotype rather than as a uniform prognostic marker or as evidence of a causal effect of the valve lesion.

## Conclusions

Among adults admitted to the intensive care unit for a non-cardiac primary diagnosis, velocity-defined moderate aortic stenosis identified during that admission was independently associated with higher 90-day mortality, with a dose–response gradient across severity grades. In exploratory analyses the association was numerically concentrated in the first weeks after the echocardiogram and in patients with reduced ejection fraction, and was small in those with preserved function, although neither pattern was supported by a significant formal test. These observational data identify velocity-defined moderate aortic stenosis as a marker of a higher-risk phenotype in the critically ill; they do not establish that moderate valvular obstruction itself causes the excess mortality, which may instead reflect the interaction of the valve lesion with a vulnerable myocardium, the associated cardiac damage, flow-dependent misclassification, and residual confounding. Moderate stenosis identified in this setting may therefore warrant closer surveillance and structured follow-up rather than, on current evidence, valve intervention; whether earlier treatment benefits this population requires dedicated prospective study.

## Supplementary Information

Additional file 1: Supplementary methods and Tables S1–S5. Detailed data sources and cohort definition, the full multivariable model (Table S1), covariate balance before and after propensity-score matching (Table S2), the velocity–gradient cross-classification (Table S3), the concordance, sensitivity, subgroup, and time-stratified hazard ratios (Table S4), and definitions of all study variables (Table S5).

## Data Availability

The data that support the findings of this study are available from PhysioNet (https://physionet.org) to credentialed users who have completed the required training and signed the data use agreement: MIMIC-IV (https://physionet.org/content/mimiciv/3.1/) and MIMIC-IV-ECHO (https://physionet.org/content/mimic-iv-echo/1.0/). The analytic code and the exact cohort-extraction queries used in this study are openly available in the Zenodo repository, https://doi.org/10.5281/zenodo.20588072.

## Abbreviations

CI: Confidence interval
ICD: International Classification of Diseases
ICU: Intensive care unit
LVEF: Left-ventricular ejection fraction
MIMIC-IV: Medical Information Mart for Intensive Care IV
MIMIC-IV-ECHO: MIMIC-IV echocardiogram-matched subset
OR: Odds ratio
HR: Hazard ratio
sHR: Subdistribution hazard ratio
SMD: Standardized mean difference
SOFA: Sequential Organ Failure Assessment
STROBE: Strengthening the Reporting of Observational Studies in Epidemiology
