# Supplementary material for: Moderate aortic stenosis identified during intensive care admission for a non-cardiac primary diagnosis and 90-day mortality: a retrospective cohort study using the MIMIC-IV echocardiography database
Source: BMC Cardiovasc Disord. 2026 Aug 13;26:1052. doi: 10.1186/s12872-026-06468-3 (PMC13750021; doi:10.1186/s12872-026-06468-3)
Supplement: Supplementary file 1 [file 12872_2026_6468_MOESM1_ESM.docx]

**Supplementary Material**

***Moderate Aortic Stenosis Identified During Intensive Care Admission for a Non-Cardiac Primary Diagnosis and 90-Day Mortality: A Retrospective Cohort Study Using the MIMIC-IV Echocardiography Database***

**Contents.** Supplementary Methods (data sources and version linkage; definition of the non-cardiac primary cohort; grading of aortic stenosis; covariate and outcome definitions; sensitivity, subgroup, and time-stratified analyses; software). Table S1, full multivariable Cox model. Table S2, covariate balance before and after propensity-score matching. Table S3, velocity–gradient cross-classification. Table S4, concordant grading, sensitivity, subgroup, and time-stratified hazard ratios. Table S5, data sources and definitions of study variables.

**Supplementary Methods**

***Data sources and version linkage***

Clinical data were drawn from MIMIC-IV version 3.1 and structured echocardiographic measurements from the MIMIC-IV-ECHO module version 1.0, which provides measurements from echocardiograms performed between 2008 and 2022 in patients matched to the MIMIC-IV Clinical Database. Both resources are cited here in full, so that this file can be read independently of the main reference list: Johnson AEW, Bulgarelli L, Shen L, Gayles A, Shammout A, Horng S, et al. MIMIC-IV, a freely accessible electronic health record dataset. Sci Data. 2023;10(1):1; and Gow B, Pollard T, Greenbaum N, Moody B, Han A, Waks JW, et al. MIMIC-IV-ECHO: echocardiogram matched subset (version 1.0). PhysioNet. 2026. doi:10.13026/nrjh-5r77. Because the de-identified patient identifiers and the per-patient date-shift offsets are held constant, echocardiographic studies were linked to hospitalizations and ICU stays at the patient and admission level.

***Definition of the non-cardiac primary cohort***

The primary cohort comprised patients whose primary hospital diagnosis was non-cardiac, so that aortic stenosis was identified on an echocardiogram obtained during the evaluation of the acute illness rather than being the reason for admission. A cardiac primary diagnosis was defined from the principal discharge diagnosis code as a heart-specific condition—ischaemic heart disease, heart failure, cardiac arrhythmia, valvular and rheumatic heart disease, pericardial and myocardial disease, or cardiac arrest. Cerebrovascular and other vascular diagnoses were treated as non-cardiac, because in such admissions aortic stenosis is likewise not the reason for admission. Pre-admission ambulatory records and prior outpatient echocardiograms are not captured in the database, so we could not determine whether the valve lesion had already been diagnosed before the index admission; the exposure is described throughout as aortic stenosis identified during an ICU admission for a non-cardiac primary diagnosis, which characterizes the circumstances of detection and does not assert that the lesion was previously unknown. The full cohort of all critically ill adults, including those with a cardiac primary diagnosis, was analysed as a sensitivity analysis (Table S4).

***Grading of aortic stenosis***

Severity was graded from the peak aortic jet velocity according to the American Society of Echocardiography and the European Association of Cardiovascular Imaging, whose recommendations are cited here in full for the same reason: Baumgartner H, Hung J, Bermejo J, Chambers JB, Edvardsen T, Goldstein S, et al. Recommendations on the echocardiographic assessment of aortic valve stenosis: a focused update from the European Association of Cardiovascular Imaging and the American Society of Echocardiography. Eur Heart J Cardiovasc Imaging. 2017;18(3):254-75. Because the aortic valve area, the left-ventricular outflow-tract velocity–time integral, and the dimensionless index are not available in the database, flow-independent grading could not be performed; the peak velocity is flow-dependent and may be increased by high-output states or reduced by a low stroke volume. To address this, the mean transvalvular gradient—recorded in a subset of studies—was used to grade severity concurrently in the concordance analysis, and variables reflecting the systemic circulatory state (heart rate, temperature, and haemoglobin concentration) were included as covariates. These variables are indirect surrogates for the circulatory conditions that raise or lower transvalvular velocity rather than measurements of transvalvular flow itself, which would require the stroke volume or the left-ventricular outflow-tract velocity–time integral; they therefore mitigate but cannot eliminate flow-dependent misclassification.

***Covariate and outcome definitions***

Covariates and outcomes are defined in Table S5. Three continuous indirect markers of the systemic circulatory state (first-day minimum haemoglobin, mean heart rate, and maximum temperature) were included to mitigate flow-dependent misclassification of stenosis severity; they are surrogates for conditions that alter transvalvular flow, not direct measures of flow. The primary outcome was all-cause mortality within 90 days of the baseline echocardiogram, analysed over a fixed follow-up window; secondary outcomes were in-hospital mortality censored at discharge and 28-day mortality. For the in-hospital outcome we additionally fitted a Fine–Gray subdistribution-hazard model with the same covariates, treating live hospital discharge as a competing event, to address informative censoring at discharge.

***Sensitivity, subgroup, and time-stratified analyses***

The concordance analysis re-graded moderate and severe stenosis using concurrent peak-velocity and mean-gradient criteria, both within the gradient-available stratum and against the full no-stenosis reference. The non-hyperdynamic subset (previously termed the low-flow subset) excluded patients with a heart rate above 100 beats per minute, a temperature above 38 °C, or a haemoglobin below 9 g/dL; the restriction removes the tachycardic, febrile, and anaemic states that raise transvalvular velocity, and is therefore more precisely described as non-hyperdynamic than as low-flow, since the criteria are indirect surrogates rather than measures of transvalvular flow. Additional analyses restricted the cohort to echocardiograms performed within 24 hours of ICU admission, refitted the primary model without the vasopressor and mechanical-ventilation terms, and extended the analysis to the full cohort including cardiac admissions. To assess over-adjustment and residual confounding, we also fitted a parsimonious model (age, sex, SOFA score, and Charlson index), a model adding heart failure, and a model adding heart failure and the left-ventricular ejection fraction. Two subgroups were pre-specified, restricted to patients with a recorded ejection fraction: preserved (≥50%) and reduced (<50%) left-ventricular ejection fraction; the difference between strata was assessed with a multiplicative stenosis-grade-by-ejection-fraction interaction term evaluated by a likelihood-ratio test. All subgroup and sensitivity analyses were exploratory and unadjusted for multiple comparisons; the interaction test was treated as the formal assessment of effect modification, and stratum-specific estimates as descriptive. Although the Schoenfeld test of the proportional-hazards assumption for aortic stenosis grade was not significant (P = 0.32), a post-hoc time-stratified analysis estimated hazard ratios separately for days 0–14 and days 15–90 (the latter left-truncated at day 14) to characterize the time-course of risk by grade.

***Software***

Analyses were performed in R version 4.5.2 (survival, rms, MatchIt, cmprsk, sandwich, lmtest, and EValue packages); the cohort-construction queries were executed in Google BigQuery. The E-value was computed for a common outcome. Propensity-score matching used 1:4 nearest-neighbour matching without replacement on the logit of the propensity score with a calliper of 0.2 standard deviations; the hazard ratio in the matched sample was estimated with a Cox model using cluster-robust standard errors.

**Table S1.** Full multivariable Cox proportional-hazards model for 90-day all-cause mortality in the primary (non-cardiac) cohort (n = 12,878).

| **Variable** | **Hazard ratio (95% CI)** | **P value** |
| --- | --- | --- |
| ***Aortic stenosis severity (reference: no stenosis)*** | | |
| Aortic sclerosis | 1.169 (1.070–1.277) | <0.001 |
| Moderate stenosis | 1.330 (1.135–1.558) | <0.001 |
| Severe stenosis | 1.606 (1.272–2.027) | <0.001 |
| ***Covariates*** | | |
| Age, per year | 1.015 (1.012–1.018) | <0.001 |
| Female sex | 0.972 (0.909–1.040) | 0.41 |
| SOFA score, per point | 1.150 (1.137–1.163) | <0.001 |
| Charlson comorbidity index, per point | 1.189 (1.173–1.205) | <0.001 |
| Hypertension | 0.846 (0.783–0.915) | <0.001 |
| Atrial fibrillation | 1.013 (0.944–1.088) | 0.72 |
| Diabetes mellitus | 0.747 (0.694–0.805) | <0.001 |
| Chronic kidney disease | 0.679 (0.625–0.736) | <0.001 |
| Vasopressor, ICU day 1 | 0.898 (0.825–0.979) | 0.014 |
| Mechanical ventilation, ICU day 1 | 1.245 (1.148–1.350) | <0.001 |
| Haemoglobin, minimum, per g/dL | 0.944 (0.930–0.959) | <0.001 |
| Heart rate, mean, per beat/min | 1.016 (1.014–1.018) | <0.001 |
| Temperature, maximum, per °C | 0.754 (0.719–0.791) | <0.001 |

*Hazard ratios are mutually adjusted estimates from a single Cox model; no stenosis is the reference for the exposure. The model is specified to estimate the association of aortic stenosis severity with mortality. The coefficients for the remaining covariates are conditional associations within this model and are subject to the “Table 2 fallacy”: in particular, the apparently protective associations for hypertension, diabetes, chronic kidney disease, vasopressor use, and maximum temperature reflect statistical adjustment (collinearity with the SOFA score and the Charlson index—which already incorporate vasopressor use and other organ-support information—and collider or mediation structures) rather than causal effects and must not be interpreted as such. The proportional-hazards assumption was not rejected for aortic stenosis grade (Schoenfeld test, P = 0.32); the moderate-stenosis hazard was nonetheless numerically concentrated early on post-hoc time-stratified analysis (Table S4). CI, confidence interval; ICU, intensive care unit; SOFA, Sequential Organ Failure Assessment.*

**Table S2.** Covariate balance before and after 1:4 propensity-score matching of moderate stenosis with no stenosis (non-cardiac cohort).

| **Covariate** | **Moderate**  **(before)** | **No stenosis**  **(before)** | **SMD**  **(before)** | **Moderate**  **(after)** | **No stenosis**  **(after)** | **SMD**  **(after)** |
| --- | --- | --- | --- | --- | --- | --- |
| Age, years | 77.95 | 65.59 | 0.884 | 77.95 | 78.13 | −0.017 |
| Female sex | 0.470 | 0.467 | 0.005 | 0.470 | 0.476 | −0.012 |
| SOFA score | 5.74 | 5.05 | 0.190 | 5.74 | 5.83 | −0.028 |
| Charlson index | 7.22 | 5.54 | 0.611 | 7.22 | 7.21 | 0.003 |
| Hypertension | 0.805 | 0.666 | 0.320 | 0.805 | 0.805 | 0.002 |
| Atrial fibrillation | 0.533 | 0.327 | 0.424 | 0.533 | 0.520 | 0.026 |
| Diabetes mellitus | 0.409 | 0.322 | 0.181 | 0.409 | 0.401 | 0.016 |
| Chronic kidney disease | 0.426 | 0.259 | 0.356 | 0.426 | 0.412 | 0.028 |
| Vasopressor, ICU day 1 | 0.353 | 0.276 | 0.167 | 0.353 | 0.352 | 0.003 |
| Mechanical ventilation, ICU day 1 | 0.316 | 0.332 | −0.034 | 0.316 | 0.337 | −0.044 |

*Values are means (proportions for binary variables). Matching used 1:4 nearest-neighbour matching without replacement on the logit propensity score with a calliper of 0.2 standard deviations, pairing 411 patients with moderate stenosis to 1,644 patients with no stenosis (2,055 patients in total; the before-matching no-stenosis group comprised 10,511 patients). All standardised mean differences were below 0.05 after matching (maximum 0.044). SMD, standardised mean difference; SOFA, Sequential Organ Failure Assessment.*

**Table S3.** Cross-classification of velocity-based and mean-gradient–based aortic stenosis grade among patients with a recorded mean gradient (non-cardiac cohort).

| **Velocity-based grade** | **Gradient: none/mild** | **Gradient: moderate** | **Gradient: severe** | **No gradient recorded** |
| --- | --- | --- | --- | --- |
| No stenosis | 380 | 0 | 0 | 10,131 |
| Aortic sclerosis | 1,034 | 41 | 1 | 721 |
| Moderate stenosis | 44 | 341 | 4 | 22 |
| Severe stenosis | 0 | 22 | 136 | 1 |

*A mean transvalvular gradient was recorded in 2,003 of the 12,878 patients. Gradient grade bands were none/mild <20 mmHg, moderate 20–39 mmHg, and severe ≥40 mmHg. Among the 389 patients with velocity-defined moderate stenosis who had a recorded gradient, 341 (88%) were concordant by gradient. Adjusted hazard ratios under concordant grading are given in Table S4.*

**Table S4.** Adjusted hazard ratios for mortality under concordant grading, sensitivity analyses, pre-specified subgroups, and a time-stratified analysis (non-cardiac cohort).

| **Analysis** | **n** | **Aortic sclerosis**  **(95% CI)** | **Moderate stenosis**  **(95% CI)** | **Severe stenosis**  **(95% CI)** |
| --- | --- | --- | --- | --- |
| ***Concordant velocity–gradient grading (90-day mortality, HR)*** | | | | |
| Gradient-available stratum | 857 | — | 1.29 (1.00–1.67) | 1.51 (1.09–2.09) |
| Full no-stenosis reference | 10,988 | — | 1.36 (1.15–1.61) | 1.63 (1.27–2.09) |
| ***Sensitivity analyses (90-day mortality, HR)*** | | | | |
| Parsimonious model (age, sex, SOFA, Charlson) | 12,878 | 1.10 (1.01–1.20) | 1.22 (1.05–1.43) | 1.50 (1.19–1.90) |
| Adjusted for heart failure | 12,878 | 1.17 (1.07–1.28) | 1.35 (1.15–1.58) | 1.62 (1.28–2.05) |
| Adjusted for heart failure and ejection fraction | 10,324 | 1.14 (1.03–1.26) | 1.26 (1.05–1.52) | 1.75 (1.35–2.26) |
| Non-hyperdynamic subset | 6,000 | 1.17 (1.00–1.37) | 1.21 (0.93–1.57) | 1.72 (1.20–2.49) |
| Echocardiogram within 24 h of admission | 9,513 | 1.17 (1.05–1.29) | 1.30 (1.08–1.56) | 1.41 (1.08–1.84) |
| Without vasopressor and ventilation terms | 12,878 | 1.17 (1.07–1.27) | 1.31 (1.12–1.54) | 1.57 (1.24–1.98) |
| Full cohort including cardiac admissions | 17,679 | 1.11 (1.03–1.21) | 1.38 (1.21–1.59) | 1.52 (1.26–1.84) |
| Fine–Gray competing-risks model (in-hospital death, sHR) | 12,878 | 1.23 (1.10–1.38) | 1.47 (1.19–1.81) | 1.41 (1.00–1.99) |
| ***Pre-specified subgroups (90-day mortality, HR)*** | | | | |
| Preserved LVEF (≥50%) | 7,841 | 1.12 (1.00–1.25) | 1.10 (0.87–1.39) | 1.58 (1.16–2.15) |
| Reduced LVEF (<50%) | 2,483 | 1.13 (0.91–1.38) | 1.47 (1.10–1.97) | 1.91 (1.21–3.00) |
| ***Time-stratified analysis (post hoc, HR)*** | | | | |
| Days 0–14 | 12,878 | 1.22 (1.09–1.37) | 1.58 (1.30–1.93) | 1.51 (1.10–2.08) |
| Days 15–90 | 10,865 | 1.09 (0.95–1.25) | 1.04 (0.80–1.36) | 1.69 (1.20–2.37) |

*All estimates are hazard ratios from Cox proportional-hazards models adjusted for the covariates listed in the Methods, with no stenosis as the reference; em dashes denote categories not defined for a given analysis. The gradient-available stratum compares concordantly graded moderate and severe stenosis with concordant no stenosis among patients with a recorded gradient; the full no-stenosis reference compares the same concordant groups with all patients without stenosis. The full-cohort analysis adds the cardiac-diagnosis covariate, and the without-support analysis omits the vasopressor and mechanical-ventilation covariates. The Fine–Gray row reports covariate-adjusted subdistribution hazard ratios (sHR) for in-hospital death, with live hospital discharge treated as a competing event; severe stenosis was based on few in-hospital events and its confidence interval reaches the null. The time-stratified analysis characterizes the grade-specific time-course of risk (the Schoenfeld test for aortic stenosis grade was not significant, P = 0.32): the days 15–90 estimate is left-truncated at day 14. The moderate-stenosis hazard was numerically concentrated in days 0–14, whereas the severe-stenosis hazard was sustained; this time-stratified comparison was post hoc and is exploratory. The parsimonious model included age, sex, the SOFA score, and the Charlson index; the heart-failure and ejection-fraction models added those covariates to the primary model. The ejection-fraction subgroups were restricted to the 10,324 patients with a recorded ejection fraction (ejection fraction was unavailable in 2,554). Within the preserved-ejection-fraction stratum the numbers of patients (and 90-day deaths) by grade were: no stenosis 6,282 (1,494), aortic sclerosis 1,218 (380), moderate stenosis 243 (77), and severe stenosis 98 (42); within the reduced-ejection-fraction stratum they were: no stenosis 2,082 (726), aortic sclerosis 271 (106), moderate stenosis 94 (50), and severe stenosis 36 (20). The multiplicative interaction between aortic-stenosis grade and ejection-fraction category was not statistically significant (P = 0.21, likelihood-ratio test, 3 degrees of freedom); the difference between the two strata is therefore an exploratory numerical pattern, and these data neither establish nor exclude effect modification by ejection fraction. The non-hyperdynamic subset (heart rate ≤100 beats/min, temperature ≤38 °C, haemoglobin ≥9 g/dL) was termed the low-flow subset in the previous version; the criteria are indirect surrogates for the systemic circulatory state rather than measures of transvalvular flow; within this subset of the non-cardiac cohort there were 62 deaths among patients with moderate stenosis. Three further estimates quoted in the main text are not tabulated separately: applying the same non-hyperdynamic restriction to the full cohort including cardiac admissions gave a moderate-stenosis hazard ratio of 1.38 (1.12–1.71), and in the full cohort the ejection-fraction pattern was similar to that shown here (moderate stenosis: reduced ejection fraction 1.64, preserved ejection fraction 1.11). All sensitivity and subgroup analyses were exploratory and unadjusted for multiple comparisons. CI, confidence interval; HR, hazard ratio; LVEF, left-ventricular ejection fraction.*

**Table S5.** Data sources and definitions of study variables.

| **Item** | **Source and definition** |
| --- | --- |
| Clinical database | MIMIC-IV version 3.1 (hospital, ICU, and derived modules), Beth Israel Deaconess Medical Center. |
| Echocardiographic database | MIMIC-IV-ECHO version 1.0, a structured echocardiogram-matched module containing measurements from echocardiograms performed between 2008 and 2022 in patients matched to the MIMIC-IV Clinical Database; provides peak aortic jet velocity, mean transvalvular gradient, and left-ventricular ejection fraction. |
| Non-cardiac primary cohort | Patients whose primary hospital diagnosis fell outside the heart-specific ICD categories—ischaemic heart disease, heart failure, cardiac arrhythmia, valvular and rheumatic heart disease, pericardial and myocardial disease, and cardiac arrest. Cerebrovascular and other vascular diagnoses were classified as non-cardiac. |
| Aortic stenosis severity | Graded from peak aortic jet velocity per the American Society of Echocardiography and the European Association of Cardiovascular Imaging: none <2.0, aortic sclerosis or mild stenosis 2.0–2.9, moderate 3.0–3.9, severe ≥4.0 m/s. Velocities outside 0.5–8.0 m/s were treated as invalid. |
| Mean transvalvular gradient | Used for the concordance analysis; grade bands none/mild <20, moderate 20–39, severe ≥40 mmHg, within a plausible range of 0–150 mmHg. |
| Prior aortic valve replacement | Identified from procedure or diagnosis codes for surgical or transcatheter aortic valve replacement, or from a prosthetic-valve descriptor on the echocardiographic report; such patients were excluded. |
| SOFA score | First-day Sequential Organ Failure Assessment score from the MIMIC-IV derived first-day severity tables. |
| Charlson comorbidity index | From the MIMIC-IV derived comorbidity table. |
| Comorbidities | Hypertension, atrial fibrillation, diabetes mellitus, chronic kidney disease, heart failure, and chronic pulmonary disease, defined from recorded ICD-9/ICD-10 diagnoses and the standard MIMIC-IV derived comorbidity definitions. |
| Organ support | Vasopressor administration and invasive mechanical ventilation on the first ICU day from the MIMIC-IV derived treatment tables. |
| Indirect markers of circulatory state | First-day minimum haemoglobin, mean heart rate, and maximum temperature from the MIMIC-IV derived first-day laboratory and vital-sign tables. These are indirect surrogates for the circulatory conditions that alter transvalvular velocity, not direct measures of transvalvular flow. |
| Mortality | All-cause death from the recorded date of death; the primary outcome was death within 90 days of the baseline echocardiogram over a fixed follow-up window. |

*ICD, International Classification of Diseases; ICU, intensive care unit; LVEF, left-ventricular ejection fraction; SOFA, Sequential Organ Failure Assessment. Comorbidity and organ-support variables follow the standard MIMIC-IV derived-table definitions; the exact code lists used in cohort extraction are available from the corresponding author on request.*
